# Partial oral antibiotic therapy is non-inferior to intravenous therapy in non-critically ill patients with infective endocarditis: Review and meta-analysis

**DOI:** 10.1007/s00508-020-01614-z

**Published:** 2020-02-10

**Authors:** Richard Rezar, Peter Jirak, Michael Lichtenauer, Christian Jung, Alexander Lauten, Uta C. Hoppe, Bernhard Wernly

**Affiliations:** 1 Clinic of Internal Medicine II, Department of Cardiology, Paracelsus Medical University of Salzburg, Müllner Hauptstraße 48, 5020 Salzburg, Austria; 2 Division of Cardiology, Pulmonology and Vascular Medicine, Medical Faculty, University of Düsseldorf, Düsseldorf, Germany; 3 Department of Cardiology, University Heart Center Charité Berlin, German Center for Cardiovascular Research (DZHK), Berlin, Germany

**Keywords:** Endocarditis, Bacterial infection, Partial oral therapy, POT, Outpatient antibiotic therapy

## Abstract

**Background:**

Antimicrobial therapy is a cornerstone in the treatment of infective endocarditis (IE). Typically, intravenous (i.v.) therapy is given for 6 weeks or longer, leading to prolonged hospital stays and high costs. Several trials evaluating the efficacy of partial oral therapy (POT) have been published. This article aimed to review and meta-analyze studies comparing i.v. therapy versus POT in non-critically ill patients suffering from IE.

**Methods:**

A structured database search (based on PRISMA guidelines) regarding POT versus i.v. therapy in IE was conducted using PubMed/Medline. Primary endpoint was all-cause mortality and a secondary endpoint IE relapse. Risk rates were calculated using a random effects model (DerSimonian and Laird). Heterogeneity was assessed using the I^2^ statistics.

**Results:**

After screening 1848 studies at title and abstract levels, 4 studies were included. A total of 765 patients suffered from primary left-sided IE, whereas right-sided IE was observed in 72 patients. Mortality rates were lower in POT versus i.v. therapy (risk ratio [RR] 0.38, 95% confidence interval, confidence interval [CI] 0.20–0.74; *p* = 0.004; I^2^ 0%). IE relapse rates were similar (RR 0.63, 95% CI 0.29–1.37; *p* = 0.24; I^2^ 0%).

**Conclusion:**

Data comparing POT with standard care in IE is limited and to date only one sufficiently powered stand-alone trial exists to support its use. In this meta-analysis POT was non-inferior to i.v. therapy with respect to mortality and IE relapse in non-critically ill patients suffering from both left-sided and right-sided IE. These findings indicate that POT is a feasible treatment strategy in selected patients suffering from IE but further validation in future studies will be required.

## Introduction

Infective endocarditis (IE) still defies modern and potent anti-infective agents and leads to high death rates. Due to epidemiological changes and increasing numbers of healthcare-associated IE, mortality rates have remained unchanged in the range of more than 30% [1]. The cornerstone for successful IE treatment includes early antimicrobial therapy and source control, as well as timely cardiac surgery in selected cases [2]. Usually, i.v. therapy is given for up to 6 weeks [3]. Due to increasing healthcare costs with pressure for containment, strategies shortening the length of hospital stay are of particular interest. Heredia-Rodriguez et al. showed in their retrospective analysis that the incurred costs of IE increased from 6759.30 € per patient in 1997 to 15,489.60 € in 2008 and remained stable from that year onward [4]. Treatment plans with once daily parenteral or even oral antibiotic therapy enabling outpatient care could help to reduce costs.

Outpatient parenteral antibiotic therapy regimens (OPAT), for example with once daily i.v. administration as well as use of antibiotics with extended half-life and once weekly administration have been evaluated in acute bacterial skin and skin structure infections (ABSSSI) [5]. In this patient collective, partial oral treatment (POT) schemes are currently well established [6] and have been tested even in severe orthopedic infections [7]. Some studies evaluated the clinical potential of POT regimens in IE, and current guidelines already support its use in IE in selected patients [2, 3]. Typically, POT regimens in IE comprise an initial intravenous stabilization period and a final oral treatment phase in clinically stable patients. The rationale for POT is to shorten hospital stays in clinically stable patients with regular gastrointestinal uptake function in whom sufficient plasma levels can be achieved with orally administered antibiotics [8]. Recently in the large, randomized-controlled POET study safe outcomes for a POT regimen in patients with IE were reported [8]. This study aimed to review and meta-analyze available data of studies comparing POT versus an intravenous strategy.

## Methods

**Fig. 1:**
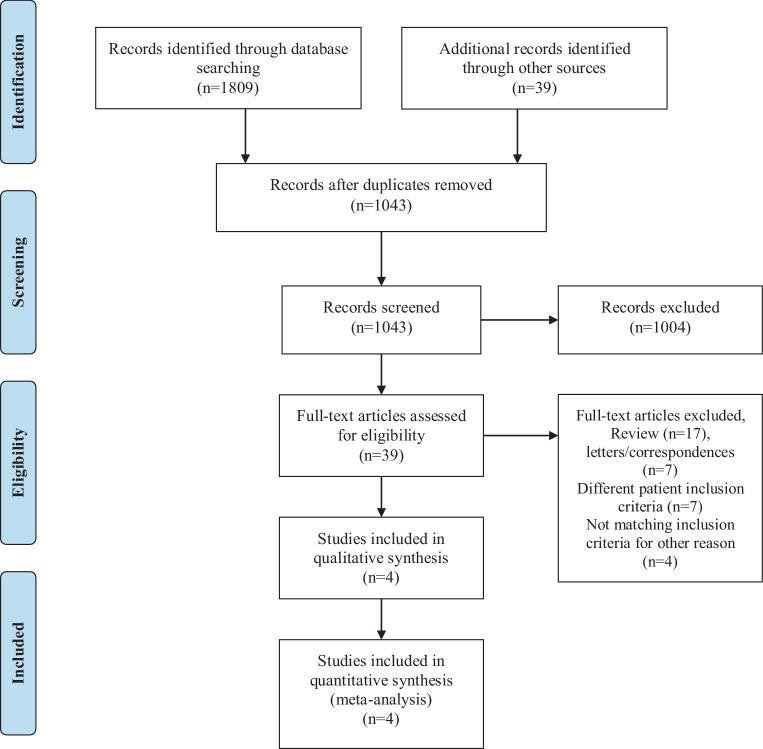
Flow diagram of the database search for screening and inclusion of studies in final meta-analysis (modified after the PRISMA [preferred reporting items for systematic reviews and meta-analyses] guidelines [9]). *n* number; *PRISMA* preferred reporting items for systematic reviews and meta-analyses

A structured database search focusing on partial oral therapy in infective endocarditis was conducted using PubMed/Medline. The following search terms were used: “infective endocarditis oral antibiotic therapy”, “infective endocarditis oral antimicrobial therapy”, “infective endocarditis outpatient antibiotic therapy”, “infective endocarditis partial oral therapy” and “infective endocarditis partial outpatient therapy”. Inclusion criteria were (1) human studies, (2) studies comparing POT versus intravenous treatment, (3) studies in the English language, (4) non-critically ill patients and (5) available baseline characteristics on POT versus intravenous treatment. The studies were screened by two researchers (BW and RR) independently. References of studies included were reviewed for further reading. The search was based on the preferred reporting items for systematic reviews and meta-analyses (PRISMA) guidelines according to the standard checklist [9]. The corresponding flow diagram is shown in Fig. 1. The primary endpoint was all-cause mortality, secondary endpoint IE relapse. Study level data were analyzed. Heterogeneity was assessed using I^2^ statistics. Risk ratios were calculated using a random effects model [10] for clinical outcomes for each individual study and consecutive pooling. Review Manager 5.3 (Version 5.3., Cochrane Collaboration, Copenhagen, Dänemark) was used for statistical computation and graphical work-up.

## Results

In total 1848 studies were screened on title and abstract level between January and October 2019. Of the studies 39 were assessed for eligibility in full text and 4 studies with a total of 788 non-critically ill patients suffering from IE were included in the final meta-analysis. In total, 70.6% of all included patients were male. The mean patient age was 57 years, whereas the mean age in the study of Heldman et al. (1996; [11]) was 35 years. All other included studies showed a comparable mean patient age in the range of 64 years.

### Narrative review

Stamboulian et al. evaluated 30 patients suffering from left-sided IE from penicillin-susceptible streptococci. Of the patients 15 were treated with i.v. ceftriaxone for 4 weeks versus another 15 patients receiving i.v. ceftriaxone for 2 weeks followed by oral amoxicillin for another 2 weeks. All patients were cured. The oral strategy avoided a total of 380 days of hospitalization [12]. In another study, Heldman et al. examined POT in i.v. drug users suffering from right-sided IE due to staphylococci. A total of 93 patients with at least 2 positive blood cultures for staphylococci were randomized in 2 groups. Patients in the oral treatment arm (*n* = 45) received ciprofloxacin and rifampicin. This group was compared to patients receiving oxacillin or vancomycin intravenously plus gentamicin for the first 5 days (*n* = 48). Only 44 patients completed treatment and follow-up. There was one treatment failure in the oral arm compared to three in the intravenous arm. Drug toxicity was observed more often in the i.v. treatment group (62% vs. 3%, mainly due to oxacillin-related increased liver enzymes) [11]. In a retrospective audit on the quality of antibiotic therapy in 66 patients with IE, Demonchy et al. observed that treatment rarely conformed with European guidelines. These findings had no impact on mortality, although a significant rate (72%) of rifampicin misuse was associated with a high prevalence of drug-related adverse effects. Antibiotics were switched from intravenous to oral administration in 29% of patients (18 ± 9 days after initiating treatment) with different therapy regimens depending on causative bacteria. They found no correlation of i.v. to oral switch and mortality in their study [13]. In a cohort study, Mzabi et al. reported outcomes of 426 patients with IE. All patients with definite or possible IE according to Duke’s criteria [14] were identified from a database. In total, 23% of all evaluated patients had a prosthetic heart valve, 12% had a permanent pacemaker and 8% had congenital heart disease. A total of 214 patients were switched to POT after an initial phase of i.v. antibiotic chemotherapy, 212 patients received i.v. only therapy. Oral treatment strategies consisted of only amoxicillin or a combination of clindamycin, fluoroquinolones, rifampicin and amoxicillin. A switch to oral therapy was not associated with increased mortality, albeit patients in the intravenous group were more ill and more often suffered from *Staphylococcus* bacteremia [15]. In the POET trial, Iversen et al. compared clinically stable patients suffering from left-sided IE. In total, 26.7% of all included patients had a prosthetic heart valve, 8.7% a permanent pacemaker and 43% suffered from previously known valvular disease. In the intravenous arm 199 patients were compared with 201 patients in the POT arm, whereas the latter were also initially treated intravenously for 10 days. Primary composite endpoint consisting of all-cause mortality, unplanned surgery, embolism or IE relapse at 6 months was similar between the two treatment groups with numerically higher mortality in the i.v. group (13 of 199 versus 7 of 201; *p* > 0.05). Adverse events from antibiotic treatment were comparable in both groups (*p* = 0.66) [8].

### Meta-analysis

**Fig. 2:**
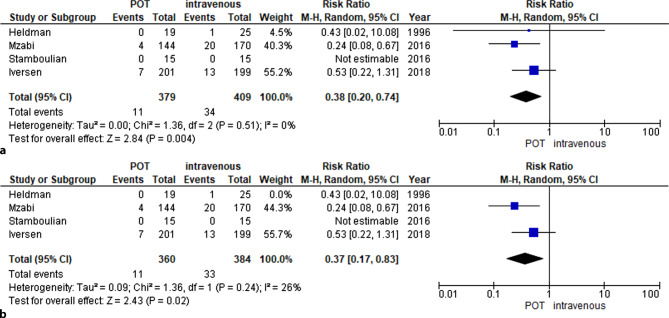
**a** Mortality was numerically lower in POT (11 of 379 vs. 34 of 409 patients). These findings were confirmed in a pooled analysis (RR 0.38, 95% CI 0.20–0.74; *p* = 0.004; I^2^ 0%). **b** Sensitivity analysis excluding studies with predominantly right-sided endocarditis confirmed aforementioned results (RR 0.37, 95% CI 0.17–0.83; *p* = 0.02; I^2^ 26%). *POT* partial oral therapy, *RR* risk ratio, *CI* confidence interval

**Fig. 3:**
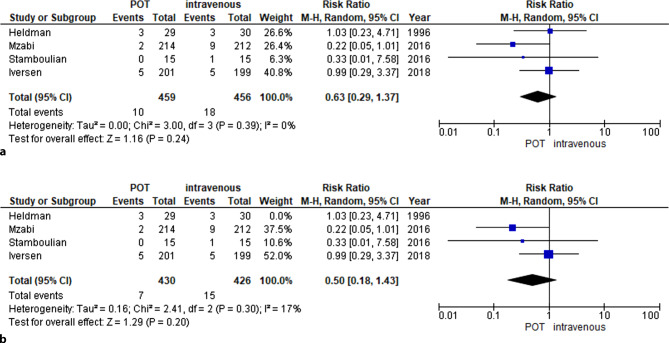
**a** Rates of relapse did not differ between POT versus intravenous treatment group (RR 0.63, 95% CI 0.29–1.37; *p* = 0.24; I^2^ 0%). **b** Sensitivity analysis excluding studies with predominantly right-sided endocarditis revealed no differences between POT versus intravenous treatment group (RR 0.50, 95% CI 0.18–1.43; *p* = 0.20; I^2^ 17%). *POT* partial oral therapy, *RR* risk ratio, *CI* confidence interval

A meta-analysis was performed comparing all available data of i.v. therapy versus POT in non-critically ill patients suffering from IE. Baseline characteristics of all included studies are given in Table 1, inclusion criteria as well as information on treatment and causative bacteria are shown in Table 2. A total of 765 patients suffered from primary left-sided endocarditis, whereas 72 patients had inter alia or solely right-sided endocarditis. All treatment regimens were adjusted to susceptibility testing. Included patients were evaluated clinically and had been classified as non-critically ill. The mortality rate was lower in POT versus i.v. strategy (RR 0.38, 95% confidence interval (CI) 0.20–0.74; *p* = 0.004; I^2^ 0% Fig. 2a). In the POT group, 11 of 379 patients died, whereas in the i.v. group 34 of 409 patients died. Endocarditis relapse rates were similar between i.v. versus oral group (RR 0.63, 95% CI 0.29–1.37; *p* = 0.24; I^2^ 0% Fig. 3a) with 10 of 459 patients in the partial oral group and 18 of 456 patients in the intravenous group experiencing a relapse. Sensitivity analysis, excluding the study from Heldman et al. evaluating only patients suffering from right-sided IE, showed similar results with respect to relapse (RR 0.50 95% CI 0.18–1.43; *p* = 0.20; I^2^ 17% Fig. 2b) and mortality (RR 0.37 95% CI 0.17–0.83; *p* = 0.02; I^2^ 26% Fig. 3b).

**Table 1:** Baseline characteristics

Study	Year	Patients		Age (years)		Left-heart		Device-associated	
–	–	POT	i.v.	POT	i.v.	POT (in %)	i.v. (in %)	POT (in %)	i.v. (in %)
Stamboulian et al. [12]	1991	15	15	59 (28–72)	63 (30–83)	100	100	0	0
Heldman et al. [11]	1996	45	48	35 ± 7	35 ± 7	0	0	0	0
Mzabi et al. [15]	2016	214	212	65 (7–98)	64 (12–93)	75	82	41	45
Iversen et al. [8]	2018	201	199	68 ± 13	67 ± 12	100	99	37	34

*RCT* randomized controlled trial, *POT* partial oral therapy, *i.v* intravenous

**Table 2:** Inclusion criteria, microorganisms and treatment regimens

Study	Inclusion criteria	Microorganisms	i.v. treatment	POT
Stamboulian et al. [12]	≥2 positive blood cultures + clinical/imaging criteria	Penicillin-susceptible streptococci	4 weeks of i.v. ceftriaxone	2 weeks i.v. ceftriaxone + 2 weeks oral amoxicillin
Heldman et al.[11]	≥2 positive blood cultures + clinical/imaging criteria, right-sided IE	Staphylococci	i.v. oxacillin or vancomycin + gentamicin	Oral ciprofloxacin + rifampicin
Mzabi et al. [15]	Possible and definite IE by Duke’s criteria	Streptococci (40%), staphylococci (30%), enterococci (12%), others (18%)	According to the nature and the susceptibility of causative microorganism; initial i.v. treatment phase	
Iversen et al. [8]	Positive Duke’s criteria	Streptococci, *Enterococcus faecalis, Staphylococcus aureus*, coagulase-negative staphylococci	According to ESC guidelines, the nature and susceptibility of causative microorganism; initial i.v. treatment phase	

*POT* partial oral therapy, *i.v* intravenous, *ESC* European Society of Cardiology, *IE* infective endocarditis

## Discussion

The meta-analysis showed non-inferiority of POT in treating non-critically ill patients with IE with respect to mortality and IE relapse. IE is a disease with stagnating incidence and mortality rates since decades [1], and a shift from occurrence in young patients suffering primarily from rheumatic valve disease to an often acute clinical course in old and sick patients with device-associated IE has been reported [16].

The first study reviewed in this analysis was published almost 30 years ago [12] and since then several changes in practice could be observed. The Duke’s criteria, first proposed in 1994, were not applied in two of the included studies [17]; however, Heldman et al. already mentioned in 1996 that using Duke’s criteria would not have changed their results, as their inclusion criteria already covered one major criterion (sustained bacteremia) and two minor criteria (fever and intravenous drug abuse) [11]. As Stamboulian et al. used similar criteria, the patient collective in the present analysis seems comparable [12]. Furthermore, imaging techniques have been improved and cross-sectional imaging as well as nuclear imaging modalities nowadays complement conventional echocardiography [18, 19]; however, this would not have affected the results of the study as bland blood cultures at follow-up were used as additional success criteria in the two earlier studies, hence minimizing the chance of undiagnosed IE-relapse [11, 12].

Further healthcare-associated IE has become more frequent [20, 21] and consequently, the spectrum of causative microorganisms has changed. The prevalence of IE due to drug-resistant bacteria as well as atypical pathogens or even fungi is increasing [22, 23]. On the other hand, highly potent antibiotic chemotherapies were developed and have enhanced the capabilities to effectively treat IE [3, 24]. Ideal antibiotic drugs inter alia show a broad spectrum bactericidal activity, sufficient tissue penetration and the possibility for oral administration. There are some orally administered substances with sufficient and constant bioavailability [25]. The different bacteria and antibiotic chemotherapies should not have a significant impact on the study results, as the included antibiotic regimens in the analysis were different, but selected according to the cultured microbial pathogen. Primary POT using resistogram-guided antibiotics combined with a short inpatient observation period could be a feasible alternative to traditional i.v.-therapy in the future; however, an initial parenteral stabilization and monitoring phase seems reasonable, as it can prevent early readmissions [26]. From our point of view, observing patients in the initial phase of IE in an inpatient setting is mandatory to rule out fulminant IE with local mechanical, infiltrative or embolic complications requiring immediate treatment [27].

With respect to the site of infection, the main proportion of patients in this meta-analysis suffered from left-sided IE, only few individuals with solely right-sided IE were included; however, sensitivity analysis did not reveal any differences. Of the studies two included patients with device-associated IE, such as pacemakers, prosthetic valves, but also individuals with congenital heart disease. Thus, POT regimens seem to not only be a possible treatment option for uncomplicated native-valve endocarditis but also selected patients with pre-existing cardiac diseases and indwelling foreign material. Future studies evaluating POT for device-associated IE are warranted.

With reference to severity of disease, in the study of Mzabi et al. certainly more patients in the i.v. group suffered from shock, acute heart failure and higher serum creatinine levels at the time of initial presentation. Nevertheless, all patients received an initial phase of i.v. therapy and a significant proportion of the initially sicker patients were included in the final oral therapy group. The latter finding applies to patients with acute heart failure and elevated serum creatinine (>100 mmol/L) but not to patients presenting with shock.

The key question regarding POT certainly is to evaluate safe inclusion criteria. In the POET trial inclusion criteria included fever below 38 °C for 2 consecutive days and a C-reactive protein value 25% below the initial peak value (or an absolute value below 20 mg/dL). These criteria seem reasonable, as they reflect antimicrobial therapy response [28]. Furthermore, individuals with abscess formation, reduced compliance, suspicion of reduced gastrointestinal absorption of antibiotics, and morbid obesity (BMI > 40 kg/m^2^) were classified as non-eligible for POT. The first three criteria are certainly out of the question. Explanatory for a suspected complicated clinical course in obese patients is the more difficult management of antibiotic therapies in overweight individuals due to often inadequate dosing, uncertain pharmacokinetic and immunomodulatory factors, as well as independent poor clinical outcome [29]. Altered pharmacokinetic aspects should also be considered in patients with right-sided congestive heart failure due to gut edema [30]. In our opinion, additional individual considerations including absence of frailty, safe home environment and professional primary medical care in temporally and locally convenient proximity of the patients’ home should be addressed. Close collaboration with general practitioners, outpatient nursing staff and finally with compliant patients is essential for therapeutic success. We propose possible criteria to select patients for POT as shown in Table 3.

**Table 3:** Criteria favoring POT or i.v. therapy respectively

Criteria favoring POT	Criteria favoring i.v. therapy
Expected sufficient patient compliance	Malcompliance, unsafe social environment
Favorable physiological conditions (formerly mobile and “healthy” patients, expected sufficient GI absorption, etc.)	Frailty, severe obesity, concomitant right-sided congestive heart failure
Afebrile patients with sufficient infection control according to clinical, laboratory and imaging parameters	Insufficient clinical improvement or continuing fever, sustained high levels of inflammatory markers, increasing size of vegetations, unstable clinical condition, local or embolic complications
Planned conservative treatment of IE	Need for cardiac surgery

*POT* partial oral therapy, *i.v*. intravenous, *GI* gastrointestinal, *IE* infective endocarditis

Formally, the death rate was even lower in this meta-analysis in POT versus i.v. therapy. Given the small patient number and high heterogeneity, we consider this primarily as a tendency towards non-inferiority of POT. Still, hospitalization could theoretically increase adverse events, as more thromboembolic events due to peripheral and central catheters and less mobilization may occur. Furthermore, hospital-acquired infections in inpatient care compared to outpatient regimen could be detrimental [31–33]. Ultimately, an intravenous antibiotic regimen should be at least equivalent with an oral treatment regimen regarding hard endpoints. The POT should be seen and used as a viable and safe alternative to parenteral regimens in clinically stable patients.

## Conclusion

In a small meta-analysis, POT was non-inferior to i.v. therapy with respect to IE relapse in non-critically ill patients suffering from both left-sided and right-sided endocarditis. The POT was furthermore associated with lower mortality rates. This finding needs to be treated with caution and validated in future studies as currently only one sufficiently powered stand-alone randomized controlled trial exists to support the use of POT in IE. The RODEO 2 trial (NCT 02701595) may provide more information about the safety of POT in patients with IE in the future; however, the present results might be interpreted as a strong signal for non-inferiority of POT in IE treatment in selected patients. Its use applies only for clinically stable patients without mechanical or embolic complications as the latter require very close clinical monitoring and i.v. administered antimicrobial therapy regimen. POT is a promising approach in modern healthcare, as it can improve quality of life, reduce hospitalization costs and free healthcare resources.

## Limitations

The comparability of different studies by different authors and countries is complicated. Furthermore, quality of evidence of included studies varies (RCT vs. cohort study). The time interval between the studies is a disadvantage with a 27-year timespan between the publication of the studies by Stamboulian et al. and Iversen et al. Table 4 gives an overview regarding level of evidence of all included studies, as well as bias and limitations of the different studies in respect to the meta-analysis.

**Table 4:** Level of evidence, bias and limitations concerning the meta-analysis

Study	Level of evidence	Risk of bias and limitations
Stamboulian et al. [12]	Single-center prospective randomized trial	Potential changes in practice due to earlier publication date; only patients with left-sided IE were included; small patient collective
Heldman et al.[11]	Single-center prospective randomized trial	Potential changes in practice due to earlier publication date; only patients with right-sided IE were included; relatively small patient collective
Mzabi et al. [15]	Retrospective cohort study	Limitation due to retrospective study design; sicker patients in the i.v. group
Iversen et al. [8]	Multi-center prospective randomized trial	–

*IE* infective endocarditis
